# Harnessing viral footprints in circulating free DNA (cfDNA) for early cancer detection: A focus on liquid‐biopsy‐based screening

**DOI:** 10.1002/ijc.70121

**Published:** 2025-09-03

**Authors:** Richard Donkor Amponsah, Emmanuel Addai Gyabaah, Moro Amidu, Zhao Cheng, Fazlur Rahman Talukdar

**Affiliations:** ^1^ Department of Medical Microbiology University of Ghana Medical School Accra Ghana; ^2^ Department of Biochemistry & Biotechnology Kwame Nkrumah University of Science & Technology Kumasi Ghana; ^3^ Cancer Research UK Cambridge Institute Li Ka Shing Centre, University of Cambridge Cambridge UK; ^4^ Cancer Research UK Cambridge Centre University of Cambridge Cambridge UK

**Keywords:** circulating free DNA (cfDNA), early cancer detection, liquid biopsy, oncogenic viruses, virus‐associated cancers

## Abstract

Viral infections play a significant role in cancer development, making detecting viral signatures a promising approach for early cancer diagnosis. Circulating free DNA (cfDNA), released into the bloodstream by tumors and other cells, has emerged as a powerful biomarker for non‐invasive cancer screening. This review explores the potential of cfDNA in detecting virus‐associated cancers through the analysis of viral footprints. It provides a comprehensive overview of the biological mechanisms underlying cfDNA release, the role of oncogenic viruses in cancer progression, and the unique viral markers that can be traced within cfDNA. By leveraging viral genetic material, cfDNA analysis offers transformative potential for the early detection of virus‐driven malignancies. Highlighting its clinical relevance, this review discusses how cfDNA‐based viral biomarker screening could significantly enhance cancer monitoring, improve prognosis accuracy, and pave the way for innovative, non‐invasive diagnostic tools and personalized cancer management strategies.

## Graphical abstract

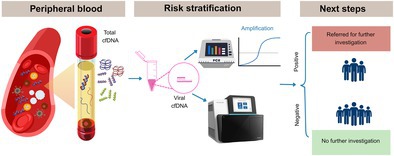

**Abbreviations**
cfDNA: circulating free DNA
cHL: Hodgkin's lymphoma
ctDNA: circulating tumor DNA
ddPCR: droplet digital polymerase chain reaction
DNA: deoxyribonucleic acid
EBV: Epstein–Barr virus
HBV: hepatitis B virus
HCC: hepatocellular carcinoma
HCV: hepatitis C virus
HPV: human papillomavirus
HTLV‐1: human T‐lymphotropic virus type 1
KSHV: Kaposi's sarcoma‐associated herpesvirus
MCPyV: Merkel cell polyomavirus
NGS: next‐generation sequencing
NPC: nasopharyngeal carcinoma
PCR: polymerase chain reaction
RNA: ribonucleic acid
Vh‐DNA: virus‐host chimera DNA
VirCAPP‐Seq: viral cancer personalized profiling by deep sequencing

## 1 INTRODUCTION

Cancer affects approximately 1 in 5 people during their lifetime, according to gender‐based analysis, indicating that 1 in 9 men and 1 in 12 women die from cancer.^1^ Infections account for 15.4% of all cancers and 9.9% are linked to viruses.^2^ Infectious agents are estimated to contribute to 17.8% of the global cancer burden, with *Helicobacter pylori*, human papillomaviruses (HPV), and hepatitis B and C viruses (HBV, HCV) accounting for the primary causes of over half of all infection‐attributable cancer cases worldwide.^3^ Additional contributors include Epstein–Barr virus (EBV), Kaposi sarcoma‐associated herpesvirus (KSHV), and human T‐cell lymphotropic virus (HTLV‐1).^4^ Understanding the role of oncogenic viruses in cancer development is complex. A single factor cannot explain it because viral infections alone are insufficient to cause cancer and typically require additional factors to contribute to tumorigenesis.^5^ Many tumor viruses can remain dormant in the host through a latent state, allowing them to take advantage of the host cell's machinery during cell division.^5^, ^6^ These viruses are broadly categorized into those directly causing cancer by encoding oncogenes and those that indirectly promote cancer through chronic inflammation and mutations.^7^, ^8^ The diversity and complexity of these oncogenic viruses, such as their distinct replication strategies, host interactions, and carcinogenic mechanisms, highlight the critical need for personalized and disease‐specific risk stratification methods to inform targeted prevention, surveillance, and treatment strategies.^9^

Virus‐associated cancer rates will continue to rise over the next four decades,^10^, ^11^ highlighting the increasing public health concern with substantial societal and economic impacts of these cancers.^12^ As such, biomarkers for early screening and prognosis are crucial. Advancements in screening, diagnosis, prevention, and treatment^11^, ^13^ contributed to a 27% decrease in cancer‐related deaths^14^ from 1991 to 2016. Although scientific advancements have provided several drugs to manage various cancers,^15^ early detection of cancer remains strongly correlated with improved clinical outcomes, including 1‐year survival rates across cancer types, except for lung and ovarian cancers.^16^, ^17^ This exception is likely due to their high genomic instability in the early stages of these cancers,^18^, ^19^ which complicates effective detection and intervention.

cfDNA has emerged as a promising non‐invasive biomarker for early cancer detection and prognosis. Present in low concentrations in the blood of healthy individuals, cfDNA levels are significantly elevated in cancer patients.^20^ A part of cfDNA, known as circulating tumor DNA (ctDNA), originates from tumor cells and carries tumor‐specific genetic alterations.^21^ Advancements in molecular biology techniques and next‐generation sequencing (NGS) have heightened interest in ctDNA as a non‐invasive biomarker for cancer detection and treatment monitoring.^21^, ^22^ Recent clinical trials have revealed the sensitivity and specificity of cfDNA as biomarkers for cancer detection, leading to the Food and Drug Authority's (FDA) approval of multigene assays for detecting genetic alterations in cfDNA.^23^ Despite its great potential, cfDNA‐based cancer tests require further research before achieving widespread clinical adoption.^22^ This review explores the possibility of cfDNA as a non‐invasive biomarker for early virus‐associated cancer detection, focusing on the detection of viral footprints in cfDNA. We also provide a comprehensive overview of the biological basis of cfDNA release, the role of oncogenic viruses in cancer progression, and the unique viral markers traced in cfDNA while addressing technological advancements and existing limitations.

## 2 ONCOGENIC VIRUSES AND THEIR ROLE IN CANCER DEVELOPMENT

**FIGURE 1:**
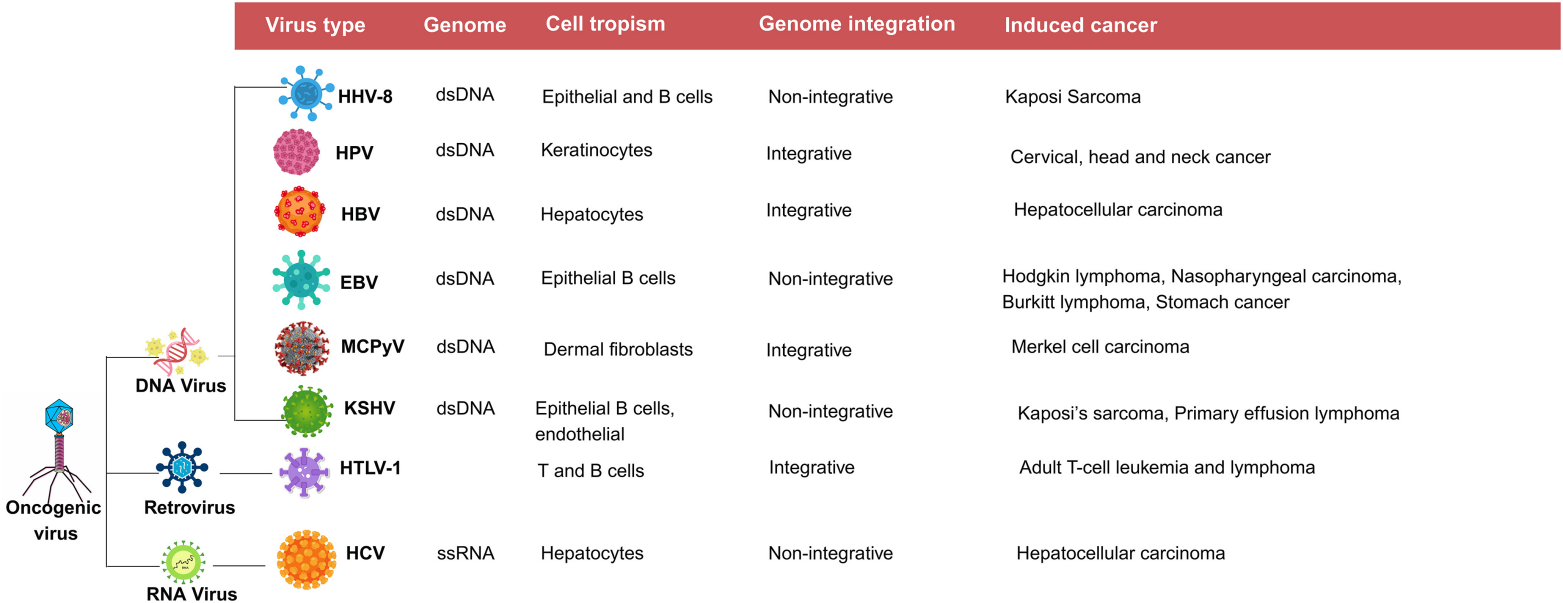
Tumor viruses and their cell tropism, host genome integration, and associated cancers. HHV‐8, human herpesvirus 8; HPV, human papillomavirus; HBV, hepatitis B virus; EBV, Epstein–Barr virus; MCPyV, Merkel cell polyomavirus; HTLV‐1, human T‐cell lymphotropic virus type 1; HCV, hepatitis C virus; KSHV, Kaposi's sarcoma‐associated herpesvirus.

Seven viruses are associated with human cancers: EBV, HPV, HCV, HBV, HTLV‐1, Merkel Cell Polyomavirus (MCPyV), and KSHV.^24^, ^25^, ^26^, ^27^ Additionally, Human Immunodeficiency Virus 1 (HIV‐1), while not directly oncogenic, is classified as an oncogenic virus due to its ability to cause immunosuppression, increasing susceptibility to carcinogenesis in the presence of other opportunistic infections.^28^ Oncogenic viruses are commonly divided into RNA and DNA tumor viruses.^28^, ^29^ RNA tumor viruses are categorized based on their mode of transformation. The known RNA tumor viruses are often retroviruses,^29^ though HCV is an exception. DNA tumor viruses are further classified by genome size and transformation mechanism. Large DNA viruses such as EBV and KSHV^29^, ^30^ transform cells through various mechanisms. In contrast, small DNA viruses such as HPV and polyomaviruses rely on viral oncogene expression to transform host cells. Small oncogenic viruses can escape apoptotic responses while keeping their host alive. This leads to the accumulation of cells with altered genomes.^29^ The classification of tumor viruses and their associated cancers is summarized in Figure 1.

Viral‐induced carcinogenesis involves multiple mechanisms, including viral integration into host genomes, oncogene activation, immune evasion, and inflammation. Oncogenic viruses can cause DNA damage and genomic instability by targeting tumor suppressors such as p53 and retinoblastoma protein (pRB).^31^ They drive cell proliferation, inhibit apoptosis, and activate oncogenes, including mitogen‐activated protein kinases, nuclear factor kappa‐light‐chain‐enhancer of activated B cells, and signal transducer and activator of transcription 3 signaling.^32^, ^33^ Additionally, they can manipulate host epigenetic machinery, altering gene expression and contributing to tumor progression.^34^ Chronic oxidative stress induced by viral infections further contributes to genomic instability and fosters neoplastic transformation.^35^ Viral integration in HBV and HPV plays a crucial role in tumor development and is linked to DNA damage.^31^ Chronic inflammation is primarily driven by the immune cell‐mediated destruction of tissue, which occurs as a result of the immune response triggered by viral infection. However, this immune response is often ineffective in controlling the infection, leading to ongoing tissue damage. For example, in HBV and HPV infections, cytotoxic T cells and inflammatory cytokines induce oxidative stress and genomic instability, driving carcinogenesis despite incomplete viral clearance.^36^ Viral immune evasion mechanisms, such as the major histocompatibility complex class 1 (MHC‐1) downregulation, exacerbate this process by prolonging infection and inflammation.^37^

## 3 BIOLOGICAL BASIS OF cfDNA IN CANCER

**FIGURE 2:**
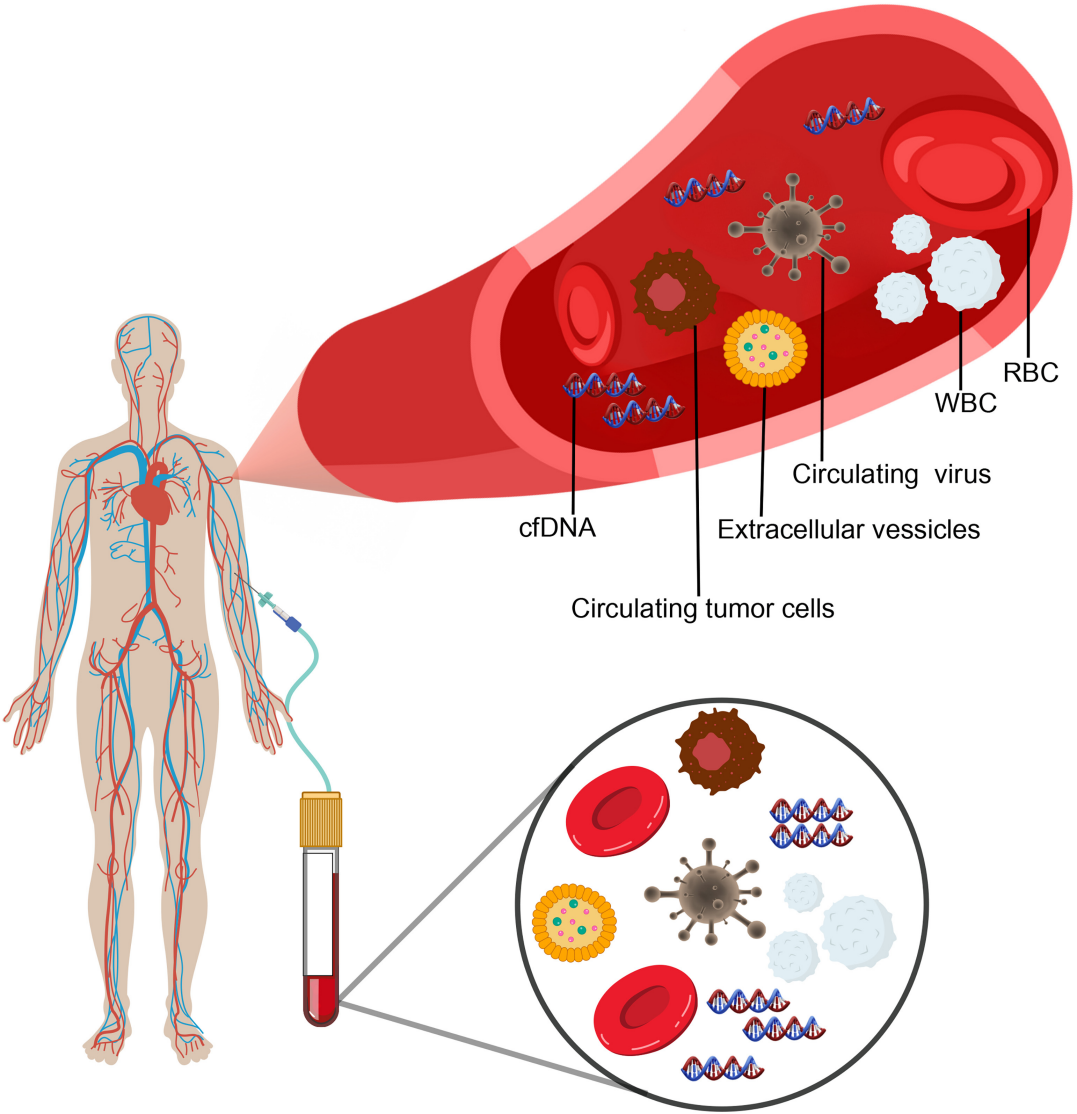
Sources of circulating free DNA in the blood. It is primarily released from the cells through apoptosis and necrosis. It is composed of healthy, viral, and tumor DNA (ctDNA).

cfDNA is released as a single‐stranded and/or double‐stranded (ds) nuclear DNA and mitochondrial DNA^38^, ^39^ into the blood^40^ of healthy and cancer patients (Figure 2). It is primarily released from cells through apoptosis, necrosis, and active secretion^41^, ^42^ and has a short half‐life ranging from 16 min to 2.5 h.^43^, ^44^ In patients with advanced lung cancer, cfDNA exists as long fragments (approximately 5 kb in size) and short fragments (approximately 170 bp), with the total amount of cfDNA being higher in cancer patients than in healthy individuals.^45^, ^46^ It contains genetic and epigenetic information that reflects cellular and tissue status, making it a tool for disease assessment.^47^, ^48^ As such, researchers are now using cfDNA analysis to screen for tumors because it consists of healthy and tumor DNA.

ctDNA, a subset of cfDNA, is shed into the bloodstream by tumor cells (Figure 2).^49^ It has gained widespread recognition as a noninvasive biomarker for cancer diagnosis and prognosis.^50^, ^51^ cfDNA holds great promise for real‐time cancer monitoring through liquid biopsy. Elevated cfDNA levels are not only observed in advanced cancer cases but have also been proposed as a diagnostic tool for breast cancer.^52^ Mead et al.^53^ highlighted the potential combination of cfDNA with other markers to offer a simple blood test as a secondary screening tool for colorectal cancers and polyps. Moreover, cfDNA end motifs were identified by analyzing the first four‐nucleotide (4‐mer) sequences at each cfDNA fragment end.^54^ These 4‐mer motifs have been demonstrated to serve as valuable biomarkers, showing promising performance for multi‐cancer detection and immunotherapy responses prediction.^47^

## 4 ORIGINS AND MECHANISMS OF cfDNA RELEASE

Cell‐free DNA (cfDNA) in the human body originates from both endogenous and exogenous sources. Endogenous contributions include DNA released during normal cellular activity, tissue damage, or programmed cell death,^54^ while exogenous sources encompass microbial or dietary DNA introduced via infection, inhalation, or digestion.^55^ A well‐characterized example is fetal DNA released into maternal circulation during pregnancy,^56^ which contributes to the cfDNA pool alongside DNA from cancerous and non‐cancerous cells.^57^ Rostami et al. demonstrated that necrosis, not apoptosis, is the primary mechanism of cfDNA release in certain cancers exposed to ionizing radiation.^58^ Nevertheless, the full spectrum of cfDNA release mechanisms remains incompletely defined.^59^

Apoptotic cell death generates mononucleosomal DNA fragments that enter the bloodstream, where nucleosomes and chromatin subunits released during DNA damage or apoptosis are detectable in circulation.^59^, ^60^ Beyond apoptosis, specialized physiological processes contribute to the cfDNA pool. For example, during erythroblast enucleation, a critical step in red blood cell maturation, the expulsion of nuclei involves cytoskeletal reorganization (e.g., actin polymerization) and establishment of nuclear polarity, followed by macrophage‐mediated engulfment of the extruded nucleus, which releases cfDNA fragments into the bloodstream.^57^, ^61^, ^62^, ^63^ Similarly, neutrophil extracellular traps (NETs)—a defense mechanism where neutrophils extrude chromatin webs to trap pathogens—release cfDNA during infections or inflammatory conditions through a process termed NETosis.^64^ These pathways highlight the complexity of cfDNA sources and signify the importance of distinguishing cellular origins when interpreting cfDNA profiles in clinical or research settings.

## 5 MECHANISTIC AND FUNCTIONAL INSIGHTS INTO cfDNA DYNAMICS

While apoptosis, necrosis, and active secretion are established sources of cfDNA,^41^, ^42^, ^55^ the biochemical pathways governing its release and stability warrant deeper elaboration. During apoptosis, nuclear DNA is cleaved into ~180‐bp fragments by caspases, which are transiently packaged into apoptotic bodies. However, subsequent nuclease degradation releases these fragments as free, unbound cfDNA into circulation, explaining why most circulating cfDNA is not vesicle‐encapsulated.^56^ In contrast, necrosis releases longer, irregular fragments (>1000 bp) due to uncontrolled membrane rupture. Active secretion via extracellular vesicles (e.g., exosomes) contributes minimally (<5% of total cfDNA) compared to apoptotic/necrotic sources.^57^ The stability of cfDNA is influenced by its association with plasma proteins (e.g., histones, albumin) that protect against nucleases, prolonging its half‐life to 16 min to 2.5 h.^38^ Beyond passive release, cfDNA may exert biological functions: upon uptake by recipient cells via endocytosis, it can activate Toll‐like receptor 9 (TLR9) mediated inflammation or integrate into genomes, potentially driving metastasis.^58^, ^59^ Tumor‐derived cfDNA, for instance, triggers neutrophil extracellular traps, fostering pro‐metastatic microenvironments.^60^ These mechanistic insights highlight the dual role of cfDNA as both a biomarker and a potential mediator of disease progression.

## 6 VIRAL FOOTPRINTS IN cfDNA: MECHANISMS AND DETECTION

When viral DNA genomes, or fragments thereof, get integrated into the host genome, they can cause cancer, which is observed in the cases of HPV in cervical and head‐and‐neck cancers, as well as with HBV in liver cancer.^61^, ^64^ This integration process is influenced by viral gene products that disrupt the DNA repair pathways.^62^, ^63^ cfDNA captures genetic material from viruses and indicates viral infection in diseases such as nasopharyngeal carcinoma (NPC).^65^, ^66^, ^67^ Garofalo et al.^68^ described how Viral Cancer Personalized Profiling by Deep Sequencing (VirCAPP‐Seq), used for profiling viral cfDNA, detects and quantifies clinically relevant viral genetic material within cfDNA. This method identifies virome composition, fragment length distribution, host genome integration sites, and viral polymorphisms.

Viral genetic material can be packaged within extracellular vesicles, such as exosomes, and released by infected cells.^69^ These vesicles carry diverse molecules, including DNA, RNA, and proteins.^70^ Recent studies have explored the potential of viral genetic signatures in cfDNA as biomarkers for virus‐related cancers. For example, HPV E7 cfDNA demonstrates high specificity for cervical cancer detection using droplet digital PCR and rapid isothermal detection methods.^71^ Cheung et al.^71^ reported the detection of HPV E7 and L1 sequences in cfDNA from cervical cancer patients with high viral load, indicating an increased risk of recurrence. In oropharyngeal squamous cell carcinoma patients, plasma HPV cfDNA levels correlated with tumor size and clinical outcome, suggesting its utility in monitoring treatment response and disease progression.^72^ Additionally, virus‐host chimera DNA (vh‐DNA) has been proposed as a novel biomarker for virus‐related cancers. The integration of viral DNA into host genomes generates the unique vh‐DNA fragments which can be enriched and utilized as a specific marker for tracking viral cancers in clinical settings, offering advantages such as reduced background interference and tumor localization.^73^

While cfDNA analysis offers a minimally invasive alternative to tissue biopsy, it has notable limitations compared to histopathology. Critically, cfDNA is tissue‐agnostic and cannot localize the anatomical source of viral DNA (e.g., distinguishing EBV in nasopharyngeal carcinoma vs. latent infection in B‐cells) or provide spatial context about disease progression (e.g., tumor‐stroma interactions, inflammatory infiltrates).^74^, ^75^ Conversely, cfDNA outperforms serological detection for non‐integrating viruses like EBV. Serology detects host antibodies (IgG/IgM), which may persist for years after resolved infections or fail to distinguish latent from active replication.^76^ In contrast, cfDNA directly quantifies cell‐free viral genomes released during lytic replication or tumor cell lysis, enabling real‐time monitoring of viral load dynamics and treatment response.^77^ For example, EBV cfDNA levels correlate with nasopharyngeal carcinoma tumor burden and predict relapses earlier than imaging or antibody titers.^78^

## 7 cfDNA AS A BIOMARKER FOR VIRUS‐ASSOCIATED CANCER DETECTION

cfDNA analysis has emerged as a promising non‐invasive alternative to traditional tissue biopsies in cancer management (Table 1). This liquid biopsy approach captures tumor heterogeneity, enables real‐time disease monitoring, and facilitates repeated sampling.^90^, ^91^ By carrying genomic, epigenomic, and fragmentomic signatures from tumor cells—including mutations, methylation patterns, and nuclease‐mediated fragmentation profiles, cfDNA facilitates early cancer detection, prognosis assessment, and prediction of therapeutic response.^92^ Advances in genomic technologies, such as NGS and digital PCR, have greatly improved the sensitivity of mutation detection in cfDNA, revolutionizing its role in clinical practice.^91^

**TABLE 1:** Summary of studies showcasing virus‐associated cancer detection and prognosis using cell‐free DNA.

Authors	Patient population	Study design	Sample size	Cancer type	CfDNA type	Detection method	Key findings	Inference
Kang et al., 2017^79^	HPV16‐ or HPV18‐positive metastatic cervical cancer patients	Retrospective study	19	Metastatic cervical cancer	HPV cfDNA	Duplex digital droplet PCR	Sensitivity: 100% Specificity: not reported	HPV cfDNA is a biomarker for detecting, monitoring, and genotyping metastatic cervical cancer for targeted therapies
Chan et al., 2017^66^	Chinese men who were 40 to 62 years of age	Prospective study	20,174	Nasopharynge al carcinoma (NPC)	EBV cfDNA	Real‐time PCR	Sensitivity: 97.1%, Specificity: 98.6% for EBV DNA in detecting nasopharyngeal carcinoma	EBV DNA analysis in plasma samples effectively screens for early asymptomatic nasopharyngeal carcinoma
Lv et al., 2024^80^	NPC Patients Undergoing Sequential Chemo‐ Radiotherapy	Prospective study	1000	Nasopharynge al carcinoma (NPC)	EBV cfDNA	Quantitative PCR‐based assay	cfEBV DNA demonstrated sensitivity to treatment changes	On‐treatment cfEBV DNA is a strong prognostic biomarker for cancer assessment
Adrian et al., 2023^81^	Patients with p16‐ positive Oropharyngeal carcinoma (OPSCC)	Prospective phase III multicenter study	136	Oropharyngeal carcinoma (OPSCC)	HPV16‐ctDNA	Real‐time quantitative PCR	Sensitivity: 79.4% pre‐treatment; post‐ treatment clearance: 74%	ctHPV16‐DNA is an independent prognostic factor in HPV‐related OPSCC
Hanna et al., 2018^82^	Patients with recurrent, metastatic HPV+ head and neck cancer	Pilot observational cohort study	22	Oropharyngeal cancer (OPC)	HPV cfDNA	Droplet digital (dd) PCR	HPV cfDNA levels strongly correlate with total tumor burden, disease site, and prognosis	cfHPV DNA levels are a promising biomarker for assessing tumor burden in metastatic HPV‐ positive oropharyngeal cancer
Reder et al., 2020^72^	Patients with HPV16‐driven Oropharyngeal carcinoma (OPSCC)	Pilot observational study	30	Oropharyngeal carcinoma (OPSCC)	HPV cfDNA	Real‐time quantitative PCR (qPCR)	Plasma cfHPV‐ DNA detection correlates with disease progression in HPV‐driven OPSCC	cfHPV‐DNA in liquid biopsies is a promising biomarker in HPV‐ driven OPSCC
Cabel et al., 2021^83^	Patients with HPV‐positive Locally advanced cervical cancer (LACC)	Retrospective cohort and prospective cohort study	55	Locally advanced cervical cancer (LACC)	HPV cfDNA	Digital droplet PCR.	Sensitivity: 69% in detecting LACC before chemoradiotherapy (CRT)	HPV‐ctDNA detection was associated with tumor stage and lymph node status
Galati et al., 2022^84^	Women with HPV16‐positive cervical cancer	Retrospective study	180	Cervical squamous cell carcinoma	HPV ctDNA	Bead‐based HPV genotyping assay	Sensitivity: 74.7% Specificity: 97.8% for detecting HPV16‐ positive cervical cancer	Combined HPV markers (HPV16 ctDNA and E6 antibodies) may enhance sensitivity for early‐stage cervical cancer detection
Jeannot et al., 2021^85^	Patients with HPV16‐ or HPV18‐related cervical cancers	Prospective cohort study	94	Cervical cancers	HPV ctDNA	Digital droplet PCR	Sensitivity: 63% in detecting cancer before treatment	Patients with residual HPV ctDNA at the end of treatment have a high risk of cancer recurrence
Sivars et al., 2024^86^	Patients with locally advanced cervical cancer	Prospective observational cohort study	74	Locally advanced cervical cancer (LACC)	HPV ctDNA	Droplet digital PCR assays	ctHPV DNA was found in plasma before relapse was diagnosed	ctHPV DNA in follow‐up plasma is a promising biomarker for prognosis and early relapse detection in patients with locally advanced cervical cancer (LACC)
Shoda et al., 2017^87^	Gastric cancer patients who underwent surgery	Prospective cohort study	153	Gastric cancer	EBV cfDNA	Quantitative real‐time PCR	Sensitivity: 71.4% Specificity: 97.1% in detecting gastric cancer (GC)	Plasma EBV in EBVaGC reflects tumor burden and is a sensitive, cost‐ effective marker for monitoring tumor progression and treatment response
He et al., 2023^88^	Patients with pretreatment plasma EBV DNA	Retrospective study	1405	Nasopharynge al carcinoma (NPC)	EBV cfDNA	Real‐time quantitative PCR assay	Sensitivity: 41.5% Specificity: 99.3% in detecting nasopharyngeal carcinoma (NPC)	Pretreatment plasma EBV DNA is useful for NPC screening and patient management
Chen et al., 2021^89^	Participants seropositive for EBV VCA‐IgA and EBNA1‐ IgA in a community‐ based NPC screening	Community‐based screening cohort	1363	Nasopharynge al carcinoma (NPC)	EBV cfDNA	Real‐time quantitative PCR	Elevated plasma EBV DNA levels correlate with increased nasopharynge al carcinoma (NPC) incidence	Plasma EBV DNA monitoring complements EBV serological screening for nasopharyngeal carcinoma (NPC)

The profiles of microbial cfDNA sequences indicate their prognostic value in cancer patients.^93^ HPV cfDNA was successfully detected in 69% of patients before chemo‐radiotherapy for locally advanced cervical cancer (LACC). According to Cabel et al.,^83^ HPV‐ctDNA levels are also associated with tumor HPV copy number (*r* = 0.41, *p* < .001). EBV serological antibody testing and plasma EBV‐DNA analysis have emerged as tools in NPC screening and have contributed to early diagnosis and treatment.^94^ Ji et al.^95^ reported a sensitivity of 81.5% for EBV cfDNA in the initial screening of early NPC. Garofalo et al.^96^ also demonstrated the effectiveness of cfDNA‐based viral detection in early classical Hodgkin's Lymphoma (cHL). Using VirCAPP‐Seq, they examined the circulating virome in cHL patients and identified correlations between EBV cfDNA levels and disease characteristics. The elevated plasma EBV loads also correlated with tumor EBV status and disease severity. The VirCAPP‐Seq allows for the characterization of viral cfDNA, including virome composition, fragment length distribution, host genome integration sites, and viral polymorphisms.^68^ Hanna et al.^82^ found a relationship between total tumor burden and HPV cfDNA levels, which were detectable earlier than imaging scan changes. This suggests that monitoring HPV cfDNA levels could serve as an early indicator of cancer, allowing for earlier intervention.

Bønløkke et al.^97^ analyzed pre‐treated plasma samples from cervical cancer (CC) patients and detected HPV cfDNA in varying proportions across patient groups, with higher detection rates in patients undergoing oncological therapy. This study revealed an association between cfDNA levels, HPV16 integration status, and disease severity, suggesting that cfDNA‐based viral detection could provide valuable insights into disease progression. This could guide treatment decisions in selecting patients for adjuvant oncological therapy. Similarly, Thangarajah et al.^98^ also utilized droplet digital PCR (ddPCR) to quantify HPV cfDNA as a biomarker for monitoring molecular therapy in HPV‐driven cervical and vulvar cancers. Their findings showed that HPV cfDNA levels increase with the disease stage, highlighting its potential utility in advanced cancer management. In another study, peripheral blood samples were obtained from HBV‐related HCC patients, and the total plasma viral cfDNA was quantified using a fluorometric double‐stranded DNA assay to predict early recurrence of HBV‐related HCC.^99^ In 2019, a study found cell‐free chimeric DNA, made up of both human and HBV DNA, in the blood of 20 patients with chronic HBV infection. This chimeric DNA came from 87 sites where HBV had integrated into the human genome. Interestingly, these integration sites were often located in genes related to tumors. This suggests that the chimeric DNA in blood is a potential biomarker for diagnosing liver cancers.^100^ Zheng et al.^101^ also found that the most recurrent integration events detected in blood cfDNA had originated from tumor tissues. This concludes the potential utility of non‐invasive detection of HBV‐DNA integration as a circulating tumor marker for HBV‐related HCC.

## 8 CURRENT TECHNOLOGIES FOR DETECTING VIRAL FOOTPRINTS IN cfDNA

Liquid biopsy offers a non‐invasive alternative to tissue biopsy, addressing issues such as sampling bias and tissue heterogeneity.^102^, ^103^ cfDNA has emerged as a valuable tool, particularly in oncology, to enable minimally invasive diagnosis, treatment, and disease monitoring. However, isolating cfDNA from plasma is challenging due to its low concentration, with only a range from 0.1% to ~1% of total cfDNA contributed by tumor cells.^104^ The tumor fraction and concentration of cfDNA have a strong correlation with the tumor burden in tumors. Besides, the ratio of ctDNA in cfDNA has been associated with tumor size, stage, and metastasis in multiple studies. Effective extraction methods are crucial for achieving high yields and purities.^50^, ^103^, ^105^

Various pre‐analytical factors influence the quality and quantity of cfDNA, including matrix type, sample collection tubes, centrifugation protocols, thawing procedures, DNA isolation methods, storage of isolated DNA, quantification methods, and downstream analysis.^102^ Commercially available extraction kits utilize different binding methods, such as ethanol precipitation, spin columns, magnetic beads, and polymer‐mediated enrichment technology. The state of automation of these kits depends solely on the protocol used; however, magnetic bead‐based methods are often automated.^106^ Among these, the QIAamp kit by QIAGEN is widely considered the gold standard, although selecting the appropriate kit depends on multiple factors such as sample type, efficiency rate, and downstream applications.^102^, ^107^

Numerous methods have been developed to detect viral DNA within cfDNA, including RT‐qPCR, digital PCR, and NGS‐based approaches^108^ (Table 2), single‐molecule real‐time (SMRT),^138^ Nanopore sequencing,^139^ and Clustered Regularly Interspaced Short Palindromic Repeats (CRISPR) based diagnostics methods.^140^ These techniques allow the analysis of mutational signatures, evaluation of copy number aberrations, and assessment of tumor heterogeneity.^141^ Reverse transcription quantitative polymerase chain reaction (RT‐qPCR) demonstrated great potential for detecting viral cfDNA for cancer screening.^142^ It revealed significant differences in cfDNA concentration between breast cancer patients and healthy individuals. It also showed potential as a sensitive and specific method for viral DNA detection in cfDNA analysis. Crucitta et al.^143^ compared Droplet digital polymerase chain reaction (ddPCR) and solid digital PCR (sdPCR) for detecting epidermal growth factor receptor and rat sarcoma (RAS) mutations in cfDNA from lung and colorectal cancer patients. Although both platforms showed similar specificity, sdPCR demonstrated higher sensitivity and detected mutations in a large number of patients, even with low cfDNA abundance. NGS‐based assays, including Tagged‐amplicon Deep Sequencing (TAm‐Seq), Cancer Personalized Profiling by Deep Sequencing (CAPP‐Seq), Whole Genome Bisulfite Sequencing (WGBS‐Seq), Whole Exome Sequencing (WES), and Whole Genome Sequencing (WGS), offer a comprehensive approach to analyzing viral cfDNA fragments.^100^, ^144^ Sorbini et al.^144^ suggested that NGS allows the detection of low‐abundance viral targets within cfDNA, which is important for liquid biopsy applications. While NGS provides a broad evaluation of tumor mutations and oncogenes, its cost‐effectiveness and efficiency are influenced by assay design and coverage depth.^145^ Third‐generation sequencing technologies, such as SMRT and Nanopore sequencing, detect DNA through optical or electrical signal detection methods,^146^ revealing a substantial population of analyzable long cfDNA in plasma. Yu et al.^139^ reported that single‐molecule tissue‐of‐origin analysis using Nanopore and PacBio (SMRT) sequencing showed significantly higher liver‐derived cfDNA percentages and elevated HCC methylation scores in patients with hepatocellular carcinoma (HCC) compared to HBV carriers. By setting a threshold of three standard deviations above the mean HCC methylation scores in non‐HCC subjects, both sequencing methods achieved a sensitivity of 75% and a specificity of 100%.^139^

**TABLE 2:** Comparing technologies for detecting cfDNA.

Technology	Platform	Sensitivity	Specificity	cfDNA input	No. of targets	Type of alteration	Limitations	Clinical utility
NGS	Deep sequencing (>10,000×)	0.02%	80%–90%	2 ng	Panel	Genome‐wide copy number changes	Unable to detect rearrangements without assay customization	Non‐invasive tumor DNA detection, mutation screening, and targeted therapy selection^109^, ^110^, ^111^
	TAm‐Seq	0.02%	99.99%	0.9–20 ng	Panel	Known point mutations	Detects only known mutations	Detection of low‐ frequency cancer mutations in circulating _DNA_ ^112^
	Safe‐SeqS	0.10%	98.90%	3 ng	Panel	Known point mutations and copy number variations	Less comprehensive than WES	Accurate detection of rare mutations in circulating DNA ^113^, ^114^
	FASTSeqS	>10%	80%	5–10 ng	Panel	Genome‐wide copy number changes	Low sensitivity and specificity	Estimation of circulating DNA levels^115^, ^116^
	CAPP‐Seq	0.004%	>99.99%	32 ng	Panel	Known point mutations, copy number variations, and rearrangements	High cfDNA input; detects only known mutations	High sensitive detection of circulating _DNA_ ^117^, ^118^, ^119^
	Multiplex‐PCR NGS	>0.1%	99.6%	2–50 ng	Panel	Known point mutations	Detects only known mutations	Detection of circulating DNA in early‐stage lung cancer^120^
Digital‐PCR	ddPCR	0.10%	100%	25 ng	1–3	Known point mutations	Detects specific genomic loci; limited in multiplexing	Accurate detection of mutations in circulating DNA^121^, ^122^, ^123^
	BEAMing	0.01%	100%	1 ng	1–20	Known point mutations	Detects only known mutations	Detection of mutations (e.g., EGFR, PIK3CA) in circulating DNA and monitoring resistance in mutations (e.g., T790M)^124^, ^125^, ^126^, ^127^
Real‐Time PCR	AS‐PCR	1.00%	98%	3–50 ng	1	Known point mutations	Low sensitivity; detects known mutations	Detection of single‐base mutations or small deletions in circulating _DNA_ ^128^, ^129^, ^130^
	AS‐NEPB‐PCR	0.10%	100%	20 ng	1	Known point mutations	Detects only known point mutations	Detection of low‐ frequency mutations in cfDNA^131^
	(PNA‐LNA) PCR clamp	0.1%–1%	79%	30 ng	1	Known point mutations	Low specificity; detects only known point mutations	Detection of mutations in plasma cfDNA^132^, ^133^, ^134^
	(COLD‐PCR)	0.10%	94.9%	1–10 ng	45,294	Known point mutations	Detect limited genomic loci; limited in multiplexing	Detection of mutation in cfDNA^135^
	MS‐PCR	0.62%	100%	20–100 ng	1	Known methylation sites	Detects only specific CpG islands	Detection of methylation in liquid biopsies^136^

Abbreviations: (PNA‐LNA) PCR clamp, peptide nuclei acid‐locked nucleic acid; AS‐NEPB‐PCR, allele‐specific, non‐extendable primer blocker PCR; AS‐PCR, allele‐specific amplification; BEAMing, beads, emulsion, amplification and magnetics; CAPP‐Seq, Cancer Personalized Profiling by Deep Sequencing; COLD‐PCR, co‐amplification at lower denaturation temperature; ddPCR, droplet digital polymerase chain reaction; MS‐PCR, methylation‐ specific PCR; Safe‐SeqS, safe‐sequencing system; TAm‐Seq, tagged‐amplicon deep sequencing; WES, whole exome sequencing.^137^

In addition to NGS and digital PCR methodologies, CRISPR‐based diagnostic methods have recently emerged as innovative approaches aimed at enabling rapid, accurate testing at home. CRISPR‐based diagnostic systems leverage Cas proteins, primarily Cas12 and Cas13, for highly sensitive molecular detection. When the CRISPR‐associated protein (Cas) or single guide RNA (sgRNA) complex binds to a specific target sequence, it becomes activated, triggering nonspecific cleavage of single‐stranded DNA or RNA probes in solution. This process facilitates nucleic acid sensing through signal amplification and enables various detection methods by incorporating functionalized reporter nucleic acids, which are cleaved as a result of collateral activity.^147^ Freije et al.^148^ demonstrated the broad applicability of Cas13‐based diagnostics by identifying thousands of potential Cas13 crRNA target sites across various single‐stranded RNA (ssRNA) viruses capable of infecting humans, highlighting its potential in sensitive viral diagnostics.

## 9 RECENT ADVANCEMENTS IN cfDNA ANALYSIS AND THEIR IMPACT ON CLINICAL PRACTICE

New advances in analyzing cfDNA have enhanced its application in cancer screening and monitoring.^149^ In 2023, researchers from the David Geffen School of Medicine, University of California, and Stanford University published a comprehensive map of DNA methylation patterns using 521 healthy tissue samples from 29 organs.^150^ This map assists in pinpointing the sources of cfDNA in the blood, revealing organ‐specific damage caused by diseases. They created the deep neural network (DNN)‐based model, cfSort, which utilizes this data to accurately trace cfDNA origins, surpassing older methods. While traditional methods focus on single features like DNA methylation, a new method called Multimodal epigenetic sequencing analysis (MESA) developed by Li et al.^151^ captures four epigenetic features at once—methylation, DNA packaging, variations in DNA packaging, and protection patterns near gene regions—using a single, cost‐effective test. In tests on 690 blood samples from colorectal cancer patients, MESA outperformed older methods by combining these features, especially using new signals like DNA packaging variability and gene end‐region patterns.

Furthermore, Martin‐Alonso et al.^152^ have explored a new strategy to further enhance cfDNA detection by transiently modulating cfDNA clearance in vivo. Their approach involved using intravenous priming agents administered shortly before a blood draw. One strategy employs succinyl phosphoethanolamine–based liposomal nanoparticles that reduce the uptake of cfDNA by liver macrophages, while a complementary approach uses engineered DNA‐binding monoclonal antibodies to protect cfDNA from degradation. These priming agents were shown to increase ctDNA recovery by more than tenfold in preclinical cancer models, enabling more comprehensive tumor profiling and dramatically improving sensitivity in detecting small tumors. Adding to these developments, Esfahani et al.^153^ have introduced a novel method based on promoter fragmentation entropy (PFE) to measure the variability in the length of cfDNA fragments. Their findings revealed a positive correlation between PFE and gene expression in peripheral blood cells, and their method was effective in detecting lung cancer with an AUC of 0.83. This approach was able to evaluate responses to chemotherapy in patients with various B‐cell lymphomas.

## 10 CURRENT CHALLENGES IN cfDNA IN CANCER DETECTION

When an individual is infected, genetic material from the invading virus can be found in their blood plasma as part of cfDNA. This has proven valuable for diagnosing and monitoring virus‐associated cancer patients. However, studies have shown that viral cfDNA in plasma may not always be cancer‐associated, as cfDNA can also originate from tissue injury, inflammation, and infection unrelated to cancer.^6^ Generally, the analysis of cfDNA in blood for cancer screening faces several challenges. These include low concentrations of cfDNA, short fragment length, susceptibility to chemical damage, high sequence similarity between cancer‐derived and healthy DNA, and the need for simultaneous analysis of multiple markers.^38^, ^154^ Additionally, circulating tumor cells (CTCs) could be considered as a complementary liquid biopsy material for viral detection. CTCs are shed from primary or metastatic tumors into the bloodstream and carry genomic material from their tumor of origin.^155^ This makes them a potential source for detecting virus‐integrated tumor genomes, such as HPV‐driven cervical cancer,^156^ EBV‐associated nasopharyngeal carcinoma,^157^ and HBV‐driven hepatocellular carcinoma.^158^ CTC analysis could provide insights into viral integration events or the presence of episomal viral DNA at the single‐cell level. However, CTCs are exceedingly rare in circulation, and not all tumor cells harbor detectable viral sequences.^159^ Moreover, viral DNA within the CTC genome may exist at extremely low copy numbers, posing significant challenges for detection. Moving forward, integrating cfDNA and CTC analyses could enhance the sensitivity and specificity of viral detection in liquid biopsy, offering a more comprehensive understanding of virus‐associated cancers.

Despite significant advancements in sequencing technologies, library preparation methods, and bioinformatics tools over the past 15 years, pre‐analytical factors also limit clinical sensitivity. No dominant technology has yet addressed these challenges.^154^ For instance, Huang et al.^93^ reported that viral cfDNA detected in early‐onset breast cancer patients was not necessarily present or was present at very low levels when the disease was fully established, complicating the use of cfDNA‐based detection methods for accurate cancer diagnosis and screening. The high degree of fragmentation in viral‐derived cfDNA, as noted by Phung et al.,^160^ further complicates the enrichment and differentiation of viral sequences from the predominant human‐derived cfDNA. While plasma EBV cfDNA is readily detected in EBV‐associated malignancies, its specificity is insufficient to diagnose lymphoma, as individuals with HIV or other immunosuppression can show elevated levels of cfDNA, leading to false positives or negatives.^161^, ^162^ Moreover, interpreting results from cfDNA analysis requires careful consideration of factors such as tumor heterogeneity, variations in virus integration sites, and the potential for false positives.^163^ Conventional PCR methods, although widely used, exhibit low sensitivity for serum HPV DNA, limiting their utility as biomarkers for early detection of HPV‐related cancers.^84^, ^163^ While biological and technical challenges persist, careful optimization of viral cfDNA analysis holds promise for offering valuable information for early cancer detection.^164^

Clinical and ethical concerns regarding implementing the cfDNA‐based assay in clinical settings have been raised. One important consideration is the timing of sampling, as various factors can influence ctDNA levels, including treatments being received, concurrent inflammatory processes, and recent surgeries. For instance, when detecting minimal residual disease (MRD) post‐surgery, blood sampling should ideally occur at least 1 week after surgery, or longer for major surgeries, to allow for proper healing and to minimize background interference.^165^

Multi‐cancer early detection (MCED) tests using ctDNA are under development, with reported sensitivities ranging from 50% to 70% at high specificities.^166^ However, differences in ctDNA release and detectability across cancer types and stages present significant challenges, as tumors of different histologies and progression levels shed varying amounts of ctDNA into the bloodstream.^167^ Distinguishing between tumor‐derived and non‐tumor‐derived ctDNA can also pose significant challenges in using ctDNA in cancer detection. This can result from clonal hematopoiesis of indeterminate potential (CHIP), a phenomenon in which age‐related somatic mutations arise in non‐malignant hematopoietic cells and may be misclassified as tumor‐derived alterations, leading to false‐positive results if not properly accounted for during analysis.^168^ These tests also face challenges related to clinical utility and the potential harms of false positives.^169^ Key ethical considerations include defining screening objectives, minimizing risks, and ensuring benefits outweigh harm for participants.^169^ The non‐invasive nature of cfDNA testing may lead to broader uptake and potential direct consumer access, highlighting the need for careful implementation strategies and robust informed consent processes.^170^ While ctDNA has valuable applications in prognosis and therapy monitoring, its application in early cancer detection remains controversial. Further validation through large‐scale studies involving non‐patient populations is essential to establish its clinical relevance and address concerns about accuracy and effectiveness.^166^, ^169^

Economic considerations also play a key role in the widespread implementation of cfDNA‐based assays. HPV DNA testing for cervical cancer, beginning at age 30 and repeated at 5‐year intervals, has been identified as the most cost‐effective strategy.^171^ In cancer immunotherapy monitoring, tumor cfDNA testing can lead to significant cost savings by enabling the early evaluation of treatment efficacy.^172^ However, early cancer detection using ctDNA faces challenges such as the requirement for large blood volumes and high sequencing costs. The application of cfDNA analysis in viral‐associated cancers is evolving rapidly, offering the potential to improve diagnostic and monitoring capabilities.^6^ To achieve cost‐effective, large‐scale implementation in public health systems, integrating cfDNA testing with other blood‐based biomarkers and employing longitudinal monitoring may be necessary to overcome current limitations.

## 11 FUTURE DIRECTION

Multi‐omics integration—combining methylation, fragmentomics, and protein biomarkers—offers improved specificity. Methylation signatures at viral integration sites (e.g., HPV in cervical cancer) may distinguish malignancies from transient infections.^173^ Long‐read sequencing and AI‐driven fragmentomics could achieve >95% accuracy in discerning cancer‐derived cfDNA via end motifs and nucleosome patterns.^174^ Longitudinal monitoring through serial HPV cfDNA testing demonstrated promising results for detecting cervical cancer recurrence.^175^

Through non‐invasive blood‐based testing, ctDNA shows further promise for early cancer detection and tumor site identification. By analyzing molecular markers such as somatic mutations, DNA methylation patterns, and fragmentomic profiles, it is feasible to diagnose cancers early on^176^ and determine their tissue of origin.^177^ However, for cfDNA‐based screening to become clinically practical, additional breakthroughs are required to improve sensitivity in early‐stage disease, improve tissue‐of‐origin prediction accuracy, and minimize false‐positive rates. Integrating multi‐omics techniques, fine‐tuning machine learning algorithms, and standardizing pre‐analytical and analytical workflows will be critical. Large‐scale prospective validation studies in different, asymptomatic groups, as well as cost‐effectiveness assessments and clear therapeutic guidelines, will be required to ensure widespread uptake and responsible clinical application.

## 12 CONCLUSION

Detection of viral footprints in circulating free DNA (cfDNA) represents a significant advancement in cancer screening, particularly for virus‐associated malignancies. By identifying viral genetic material within cfDNA, this approach not only enhances the early diagnosis of cancers linked to oncogenic viruses but also provides a non‐invasive method for monitoring disease progression and treatment response. Given that viral infections contribute to approximately 15.4% of all cancers, integrating cfDNA analysis into routine clinical practice has the potential to revolutionize cancer screening and management, paving the way for personalized medicine.

Looking ahead, the future of cfDNA research is poised for remarkable breakthroughs. Innovations in sequencing technologies, such as NGS and digital PCR, are expected to enhance the sensitivity and specificity of cfDNA detection, enabling the discovery of novel biomarkers and improving our understanding of tumor heterogeneity. Additionally, combining cfDNA analysis with other biomarkers could facilitate comprehensive cancer profiling and more effective therapeutic strategies. As the field evolves, cfDNA is anticipated to play a central role in clinical oncology, driving earlier interventions and better patient outcomes.

However, to fully realize the potential of cfDNA as a biomarker for virus‐associated cancers, further rigorous research and clinical validation remain essential. Key existing challenges include pre‐analytical factors, assay validation, low concentration of cfDNA in blood samples, and the complexities of distinguishing between viral and non‐viral DNA fragments. Addressing these hurdles through focused studies will be critical to ensuring the effective clinical application of cfDNA analysis. The call to action is clear: continued investment in cfDNA research is imperative to unlock its transformative potential in cancer diagnostics and management, ultimately enhancing cancer detection and treatment strategies.

## AUTHOR CONTRIBUTIONS

**Richard Donkor Amponsah:** Conceptualization; writing – original draft; writing – review and editing; methodology; visualization; software. **Emmanuel Addai Gyabaah:** Writing – original draft; writing – review and editing. **Moro Amidu:** Writing – original draft; writing – review and editing. **Zhao Cheng:** Supervision; writing – review and editing; methodology; visualization; validation; software. **Fazlur Rahman Talukdar:** Conceptualization; supervision; writing – review and editing; methodology; visualization; validation; software.

## CONFLICT OF INTEREST STATEMENT

The authors declare no conflicts of interest.

## References

[1] World Health Organization . Global Cancer Burden Growing, Amidst Mounting Need for Services . Accessed November 28, 2024. https://www.who.int/news/item/01‐02‐2024‐global‐cancer‐burden‐growing‐‐amidst‐mounting‐need‐for‐services PMC11115397 38438207

[2] Plummer M , de Martel C , Vignat J , Ferlay J , Bray F , Franceschi S . Global burden of cancers attributable to infections in 2012: a synthetic analysis. Lancet Glob Health. 2016;4(9):e609‐e616. doi:10.1016/S2214-109X(16)30143-7 27470177

[3] Parkin DM . The Global Health burden of infection‐associated cancers in the year 2002. Int J Cancer. 2006;118(12):3030‐3044. doi:10.1002/ijc.21731 16404738

[4] Purushothaman P , Uppal T , Verma SC . Human DNA tumor viruses and oncogenesis. Animal Biotechnology. Elsevier; 2020:131‐151. doi:10.1016/B978-0-12-811710-1.00007-0

[5] Moore PS , Chang Y . Why do viruses cause cancer? Highlights of the first century of human tumour virology. Nat Rev Cancer. 2010;10(12):878‐889. doi:10.1038/nrc2961 21102637 PMC3718018

[6] Scholte LL , Bethony JM , Xian RR . Diagnosis and monitoring of virus‐associated cancer using cell‐free DNA. Curr Opin Virol. 2023;60:101331. doi:10.1016/j.coviro.2023.101331 37187125 PMC11411455

[7] Saha A , Robertson ES . Mechanisms of B‐cell oncogenesis induced by Epstein–Barr virus. J Virol. 2019;93(13):e00238‐19. doi:10.1128/JVI.00238-19 30971472 PMC6580952

[8] McGivern DR , Lemon SM . Virus‐specific mechanisms of carcinogenesis in hepatitis C virus associated liver cancer. Oncogene. 2011;30(17):1969‐1983. doi:10.1038/onc.2010.594 21258404 PMC3642622

[9] zur Hausen H . Papillomaviruses in the causation of human cancers—a brief historical account. Virology. 2009;384(2):260‐265. doi:10.1016/j.virol.2008.11.046 19135222

[10] You W , Henneberg M . Cancer incidence increasing globally: the role of relaxed natural selection. Evol Appl. 2018;11(2):140‐152. doi:10.1111/eva.12523 29387151 PMC5775494

[11] Philipson TJ , Durie T , Cong Z , Fendrick AM . The aggregate value of cancer screenings in the United States: full potential value and value considering adherence. BMC Health Serv Res. 2023;23(1):829. doi:10.1186/s12913-023-09738-4 37550686 PMC10405449

[12] Mattiuzzi C , Lippi G . Current cancer epidemiology. J Epidemiol Glob Health. 2019;9(4):217‐222. doi:10.2991/jegh.k.191008.001 31854162 PMC7310786

[13] Rahib L , Smith BD , Aizenberg R , Rosenzweig AB , Fleshman JM , Matrisian LM . Projecting cancer incidence and deaths to 2030: the unexpected burden of thyroid, liver, and pancreas cancers in the United States. Cancer Res. 2014;74(11):2913‐2921. doi:10.1158/0008-5472.CAN-14-0155 24840647

[14] Siegel RL , Miller KD , Jemal A . Cancer statistics, 2019. CA Cancer J Clin. 2019;69(1):7‐34. doi:10.3322/caac.21551 30620402

[15] Chhikara BS , Parang K . Global Cancer Statistics 2022: the trends projection analysis. Chem Biol Lett. 2023;10(1):451.

[16] Eledkawy A , Hamza T , El‐Metwally S . Precision cancer classification using liquid biopsy and advanced machine learning techniques. Sci Rep. 2024;14(1):5841. doi:10.1038/s41598-024-56419-1 38462648 PMC10925597

[17] Wise J . Diagnosing cancer early is vital, new figures show. BMJ. 2016;353:i3277. doi:10.1136/bmj.i3277 27298300

[18] Cheng Z , Mirza H , Ennis DP , et al. The genomic landscape of early‐stage ovarian high‐grade serous carcinoma. Clin Cancer Res. 2022;28(13):2911‐2922. doi:10.1158/1078-0432.CCR-21-1643 35398881 PMC7612959

[19] Cheng Z , Ennis DP , Lu B , et al. The genomic trajectory of ovarian high‐grade serous carcinoma can be observed in stic lesions. J Pathol. 2024;264(1):42‐54. doi:10.1002/path.6322 38956451

[20] Kubaczková V , Sedlaříková L , Bešše L , Almaši M , Hájek R , Ševčíková S . Potential of cell‐free circulating DNA in diagnosis of cancer. Klin Onkol. 2015;28(4):251‐259. doi:10.14735/amko2015251 26299738

[21] Stewart CM , Tsui DWY . Circulating cell‐free DNA for non‐invasive cancer management. Cancer Genet. 2018;228:169‐179. doi:10.1016/j.cancergen.2018.02.005 29625863 PMC6598437

[22] Kohler C , Barekati Z , Radpour R , Zhong XY . Cell‐free DNA in the circulation as a potential cancer biomarker. Anticancer Res. 2011;31(8):2623‐2628. 21778314

[23] Hassan S , Shehzad A , Khan SA , Miran W , Khan S , Lee Y‐S . Diagnostic and therapeutic potential of circulating‐free DNA and cell‐free RNA in cancer management. Biomedicine. 2022;10(8):2047. doi:10.3390/biomedicines10082047 PMC9405989 36009594

[24] Flippot R , Malouf GG , Su X , Khayat D , Spano J‐P . Oncogenic viruses: lessons learned using next‐generation sequencing technologies. Eur J Cancer. 2016;61:61‐68. doi:10.1016/j.ejca.2016.03.086 27156225

[25] Gaglia MM , Munger K . More than just oncogenes: mechanisms of tumorigenesis by human viruses. Curr Opin Virol. 2018;32:48‐59. doi:10.1016/j.coviro.2018.09.003 30268926 PMC6405337

[26] Galati L , Chiantore MV , Marinaro M , Di Bonito P . Human oncogenic viruses: characteristics and prevention strategies‐lessons learned from human papillomaviruses. Viruses. 2024;16(3):416. doi:10.3390/v16030416 38543781 PMC10974567

[27] White MC , Wu X , Damania B . Oncogenic viruses, cancer biology, and innate immunity. Curr Opin Immunol. 2022;78:102253. doi:10.1016/j.coi.2022.102253 36240666

[28] Isaguliants M , Bayurova E , Avdoshina D , Kondrashova A , Chiodi F , Palefsky JM . Oncogenic effects of HIV‐1 proteins, mechanisms behind. Cancers (Basel). 2021;13(2):305. doi:10.3390/cancers13020305 33467638 PMC7830613

[29] Wallace NA , Galloway DA . Viral oncogenesis. Viral Pathogenesis. Elsevier; 2016:95‐105. doi:10.1016/B978-0-12-800964-2.00008-2

[30] Krump NA , You J . Molecular mechanisms of viral oncogenesis in humans. Nat Rev Microbiol. 2018;16(11):684‐698. doi:10.1038/s41579-018-0064-6 30143749 PMC6336458

[31] Chen Y , Williams V , Filippova M , Filippov V , Duerksen‐Hughes P . Viral carcinogenesis: factors inducing DNA damage and virus integration. Cancers (Basel). 2014;6(4):2155‐2186. doi:10.3390/cancers6042155 25340830 PMC4276961

[32] Ameya G , Birri DJ . The molecular mechanisms of virus‐induced human cancers. Microb Pathog. 2023;183:106292. doi:10.1016/j.micpath.2023.106292 37557930

[33] Read SA , Douglas MW . Virus induced inflammation and cancer development. Cancer Lett. 2014;345(2):174‐181. doi:10.1016/j.canlet.2013.07.030 23941825

[34] Soliman SHA , Orlacchio A , Verginelli F . Viral manipulation of the host epigenome as a driver of virus‐induced oncogenesis. Microorganisms. 2021;9(6):1179. doi:10.3390/microorganisms9061179 34070716 PMC8227491

[35] Reuter S , Gupta SC , Chaturvedi MM , Aggarwal BB . Oxidative stress, inflammation, and cancer: how are they linked? Free Radic Biol Med. 2010;49(11):1603‐1616. doi:10.1016/j.freeradbiomed.2010.09.006 20840865 PMC2990475

[36] Iannacone M , Sitia G , Ruggeri ZM , Guidotti LG . HBV pathogenesis in animal models: recent advances on the role of platelets. J Hepatol. 2007;46(4):719‐726. doi:10.1016/j.jhep.2007.01.007 17316876 PMC1892635

[37] Guidotti LG , Chisari FV . Immunobiology and pathogenesis of viral hepatitis. Annu Rev Pathol. 2006;1:23‐61. doi:10.1146/annurev.pathol.1.110304.100230 18039107

[38] Kustanovich A , Schwartz R , Peretz T , Grinshpun A . Life and death of circulating cell‐free DNA. Cancer Biol Ther. 2019;20(8):1057‐1067. doi:10.1080/15384047.2019.1598759 30990132 PMC6606043

[39] Sun Y , An K , Yang C . Circulating cell‐free DNA. In: Strumfa I , Gardovskis J , eds. Liquid Biopsy. IntechOpen; 2019. doi:10.5772/intechopen.80730

[40] Chan AKC , Chiu RWK , Lo YMD , Clinical Sciences Reviews Committee of the Association of Clinical Biochemists . Cell‐free nucleic acids in plasma, serum and urine: a new tool in molecular diagnosis. Ann Clin Biochem. 2003;40(Pt 2):122‐130. doi:10.1258/000456303763046030 12662399

[41] Chen Z , Fadiel A , Naftolin F , Eichenbaum KD , Xia Y . Circulation DNA: biological implications for cancer metastasis and immunology. Med Hypotheses. 2005;65(5):956‐961. doi:10.1016/j.mehy.2005.04.042 16054303

[42] Teo YV , Capri M , Morsiani C , et al. Cell‐free DNA as a biomarker of aging. Aging Cell. 2019;18(1):e12890. doi:10.1111/acel.12890 30575273 PMC6351822

[43] Diehl F , Schmidt K , Choti MA , et al. Circulating mutant DNA to assess tumor dynamics. Nat Med. 2008;14(9):985‐990. doi:10.1038/nm.1789 18670422 PMC2820391

[44] Yao W , Mei C , Nan X , Hui L . Evaluation and comparison of in vitro degradation kinetics of DNA in serum, urine and saliva: a qualitative study. Gene. 2016;590(1):142‐148. doi:10.1016/j.gene.2016.06.033 27317895

[45] Zhu Y‐J , Zhang H‐B , Liu Y‐H , et al. Quantitative cell‐free circulating EGFR mutation concentration is correlated with tumor burden in advanced NSCLC patients. Lung Cancer. 2017;109:124‐127. doi:10.1016/j.lungcan.2017.05.005 28577941

[46] Leon SA , Shapiro B , Sklaroff DM , Yaros MJ . Free DNA in the serum of cancer patients and the effect of therapy. Cancer Res. 1977;37(3):646‐650. 837366

[47] Xu J , Chen H , Fan W , Qiu M , Feng J . Plasma cell‐free DNA as a sensitive biomarker for multi‐cancer detection and immunotherapy outcomes prediction. J Cancer Res Clin Oncol. 2024;150(1):7. doi:10.1007/s00432-023-05521-4 38196018 PMC10776501

[48] Lo YMD , Han DSC , Jiang P , Chiu RWK . Epigenetics, fragmentomics, and topology of cell‐free DNA in liquid biopsies. Science. 2021;372(6538):eaaw3616. doi:10.1126/science.aaw3616 33833097

[49] Abe T , Nakashima C , Sato A , et al. Origin of circulating free DNA in patients with lung cancer. PLoS One. 2020;15(7):e0235611. doi:10.1371/journal.pone.0235611 32634139 PMC7340299

[50] Wan JCM , Massie C , Garcia‐Corbacho J , et al. Liquid biopsies come of age: towards implementation of circulating tumour DNA. Nat Rev Cancer. 2017;17(4):223‐238. doi:10.1038/nrc.2017.7 28233803

[51] Ganesamoorthy D , Robertson AJ , Chen W , et al. Whole genome deep sequencing analysis of cell‐free DNA in samples with low tumour content. BMC Cancer. 2022;22(1):85. doi:10.1186/s12885-021-09160-1 35057759 PMC8772083

[52] Huang ZH , Li LH , Hua D . Quantitative analysis of plasma circulating DNA at diagnosis and during follow‐up of breast cancer patients. Cancer Lett. 2006;243(1):64‐70. doi:10.1016/j.canlet.2005.11.027 16412565

[53] Mead R , Duku M , Bhandari P , Cree IA . Circulating tumour markers can define patients with Normal colons, benign polyps, and cancers. Br J Cancer. 2011;105(2):239‐245. doi:10.1038/bjc.2011.230 21712823 PMC3142810

[54] Pham TMQ , Phan TH , Jasmine TX , et al. Multimodal analysis of genome‐wide methylation, copy number aberrations, and end motif signatures enhances detection of early‐stage breast cancer. Front Oncol. 2023;13:1127086. doi:10.3389/fonc.2023.1127086 37223690 PMC10200909

[55] Rostami A , Lambie M , Yu CW , Stambolic V , Waldron JN , Bratman SV . Senescence, necrosis, and apoptosis govern circulating cell‐free DNA release kinetics. Cell Rep. 2020;31(13):107830. doi:10.1016/j.celrep.2020.107830 32610131

[56] Jahr S , Hentze H , Englisch S , et al. DNA fragments in the blood plasma of cancer patients: quantitations and evidence for their origin from apoptotic and necrotic cells. Cancer Res. 2001;61(4):1659‐1665. 11245480

[57] Bronkhorst AJ , Ungerer V , Holdenrieder S . The emerging role of cell‐free DNA as a molecular marker for cancer management. Biomol Detect Quantif. 2019;17:100087. doi:10.1016/j.bdq.2019.100087 30923679 PMC6425120

[58] García‐Olmo DC , Domínguez C , García‐Arranz M , et al. Cell‐free nucleic acids circulating in the plasma of colorectal cancer patients induce the oncogenic transformation of susceptible cultured cells. Cancer Res. 2010;70(2):560‐567. doi:10.1158/0008-5472.CAN-09-3513 20068178

[59] Niu Z , Tang W , Liu T , et al. Cell‐free DNA derived from cancer cells facilitates tumor malignancy through toll‐like receptor 9 signaling‐triggered Interleukin‐8 secretion in colorectal cancer. Acta Biochim Biophys Sin (Shanghai). 2018;50(10):1007‐1017. doi:10.1093/abbs/gmy104 30239551

[60] Thierry AR , El Messaoudi S , Gahan PB , Anker P , Stroun M . Origins, structures, and functions of circulating DNA in oncology. Cancer Metastasis Rev. 2016;35(3):347‐376. doi:10.1007/s10555-016-9629-x 27392603 PMC5035665

[61] Jiang Z , Jhunjhunwala S , Liu J , et al. The effects of hepatitis B virus integration into the genomes of hepatocellular carcinoma patients. Genome Res. 2012;22(4):593‐601. doi:10.1101/gr.133926.111 22267523 PMC3317142

[62] Weitzman MD , Lilley CE , Chaurushiya MS . Genomes in conflict: maintaining genome integrity during virus infection. Annu Rev Microbiol. 2010;64:61‐81. doi:10.1146/annurev.micro.112408.134016 20690823

[63] Wentzensen N , Vinokurova S , von Knebel Doeberitz M . Systematic review of genomic integration sites of human papillomavirus genomes in epithelial dysplasia and invasive cancer of the female lower genital tract. Cancer Res. 2004;64(11):3878‐3884. doi:10.1158/0008-5472.CAN-04-0009 15172997

[64] Hu Z , Zhu D , Wang W , et al. Genome‐wide profiling of HPV integration in cervical cancer identifies clustered genomic hot spots and a potential microhomology‐mediated integration mechanism. Nat Genet. 2015;47(2):158‐163. doi:10.1038/ng.3178 25581428

[65] Outinen TK , Kuparinen T , Jylhävä J , et al. Plasma cell‐free DNA levels are elevated in acute Puumala hantavirus infection. PLoS One. 2012;7(2):e31455. doi:10.1371/journal.pone.0031455 22347483 PMC3274523

[66] Chan KCA , Woo JKS , King A , et al. Analysis of plasma Epstein–Barr virus DNA to screen for nasopharyngeal cancer. N Engl J Med. 2017;377(6):513‐522. doi:10.1056/NEJMoa1701717 28792880

[67] Wuerdemann N , Jain R , Adams A , et al. Cell‐free HPV‐DNA as a biomarker for oropharyngeal squamous cell carcinoma—a step towards personalized medicine? Cancer. 2020;12(10):2997. doi:10.3390/cancers12102997 PMC7602702 33076524

[68] Garofalo A , Schroers‐Martin JG , Soo J , et al. Deep sequencing of viral cell‐free DNA for noninvasive detection of immunosuppression‐related lymphoid malignancies. Blood. 2019;134(Suppl 1):885. doi:10.1182/blood-2019-131602

[69] Bello‐Morales R , Ripa I , López‐Guerrero JA . Extracellular vesicles in viral spread and antiviral response. Viruses. 2020;12(6):623. doi:10.3390/v12060623 32521696 PMC7354624

[70] Clancy JW , D'Souza‐Schorey C . Tumor‐derived extracellular vesicles: multifunctional entities in the tumor microenvironment. Annu Rev Pathol Mech Dis. 2023;18(1):205‐229. doi:10.1146/annurev-pathmechdis-031521-022116 PMC10410237 36202098

[71] Cheung TH , Yim SF , Yu MY , et al. Liquid biopsy of HPV DNA in cervical cancer. J Clin Virol. 2019;114:32‐36. doi:10.1016/j.jcv.2019.03.005 30913520

[72] Reder H , Taferner VF , Wittekindt C , et al. Plasma cell‐free human papillomavirus oncogene E6 and E7 DNA predicts outcome in oropharyngeal squamous cell carcinoma. J Mol Diagn. 2020;22(11):1333‐1343. doi:10.1016/j.jmoldx.2020.08.002 32822851

[73] Li C‐L , Yeh S‐H , Chen P‐J . Circulating virus‐host chimera DNAs in the clinical monitoring of virus‐related cancers. Cancers (Basel). 2022;14(10):2531. doi:10.3390/cancers14102531 35626135 PMC9139492

[74] Rodríguez J , Avila J , Rolfo C , et al. When tissue is an issue the liquid biopsy is nonissue: a review. Oncol Ther. 2021;9(1):89‐110. doi:10.1007/s40487-021-00144-6 33689160 PMC8140006

[75] AbuSalah MAH , Gan SH , Al‐Hatamleh MAI , Irekeola AA , Shueb RH , Yean Yean C . Recent advances in diagnostic approaches for Epstein–Barr virus. Pathogens. 2020;9(3):226. doi:10.3390/pathogens9030226 32197545 PMC7157745

[76] Vo JH , Nei WL , Hu M , et al. Comparison of circulating tumour cells and circulating cell‐free Epstein–Barr virus DNA in patients with nasopharyngeal carcinoma undergoing radiotherapy. Sci Rep. 2016;6(1):13. doi:10.1038/s41598-016-0006-3 28442757 PMC5431344

[77] Kanakry J , Ambinder R . The biology and clinical utility of EBV monitoring in blood. Curr Top Microbiol Immunol. 2015;391:475‐499. doi:10.1007/978-3-319-22834-1_17 26428386

[78] Lo YM . Quantitative analysis of Epstein–Barr virus DNA in plasma and serum: applications to tumor detection and monitoring. Ann N Y Acad Sci. 2001;945:68‐72. doi:10.1111/j.1749-6632.2001.tb03865.x 11708496

[79] Kang Z , Stevanović S , Hinrichs CS , Cao L . Circulating cell‐free DNA for metastatic cervical cancer detection, genotyping, and monitoring. Clin Cancer Res. 2017;23(22):6856‐6862. doi:10.1158/1078-0432.CCR-17-1553 28899967 PMC7885032

[80] Lv J , Xu L‐X , Li Z‐X , et al. Longitudinal on‐treatment circulating tumor DNA as a biomarker for real‐time dynamic risk monitoring in cancer patients: the EP‐SEASON study. Cancer Cell. 2024;42(8):1401‐1414.e4. doi:10.1016/j.ccell.2024.07.001 39059389

[81] Adrian G , Forslund O , Pedersen L , Sjövall J , Gebre‐Medhin M . Circulating tumour HPV16 DNA quantification—a prognostic tool for progression‐free survival in patients with HPV‐related oropharyngeal carcinoma receiving curative chemoradiotherapy. Radiother Oncol. 2023;186:109773. doi:10.1016/j.radonc.2023.109773 37385383

[82] Hanna GJ , Supplee JG , Kuang Y , et al. Plasma HPV cell‐free DNA monitoring in advanced HPV‐associated oropharyngeal cancer. Ann Oncol. 2018;29(9):1980‐1986. doi:10.1093/annonc/mdy251 30010779

[83] Cabel L , Bonneau C , Bernard‐Tessier A , et al. HPV ctDNA detection of high‐risk HPV types during chemoradiotherapy for locally advanced cervical cancer. ESMO Open. 2021;6(3):100154. doi:10.1016/j.esmoop.2021.100154 34022731 PMC8164037

[84] Galati L , Combes J‐D , Le Calvez‐Kelm F , et al. Detection of circulating HPV16 DNA as a biomarker for cervical cancer by a bead‐based HPV genotyping assay. Microbiol Spectr. 2022;10(2):e01480‐21. doi:10.1128/spectrum.01480-21 35225653 PMC9045285

[85] Jeannot E , Latouche A , Bonneau C , et al. Circulating HPV DNA as a marker for early detection of relapse in patients with cervical cancer. Clin Cancer Res. 2021;27(21):5869‐5877. doi:10.1158/1078-0432.CCR-21-0625 34210686 PMC9401545

[86] Sivars L , Jylhä C , Crona Guterstam Y , et al. Cell‐free human papillomavirus DNA is a sensitive biomarker for prognosis and for early detection of relapse in locally advanced cervical cancer. Clin Cancer Res. 2024;30(13):2764‐2771. doi:10.1158/1078-0432.CCR-23-3941 38669077

[87] Shoda K , Ichikawa D , Fujita Y , et al. Clinical utility of circulating cell‐free Epstein–Barr virus DNA in patients with gastric cancer. Oncotarget. 2017;8(17):28796‐28804. doi:10.18632/oncotarget.15675 28430637 PMC5438692

[88] He Q , Zhou Y , Zhou J , et al. Clinical relevance of plasma EBV DNA as a biomarker for nasopharyngeal carcinoma in non‐endemic areas: a multicenter study in southwestern China. Clin Chim Acta. 2023;541:117244. doi:10.1016/j.cca.2023.117244 36746264

[89] Chen W‐J , Xu W‐N , Wang H‐Y , et al. Plasma Epstein–Barr virus DNA and risk of nasopharyngeal carcinoma in a prospective seropositive population. BMC Cancer. 2021;21(1):651. doi:10.1186/s12885-021-08408-0 34074258 PMC8168313

[90] Manoharan A , Sambandam R , Bhat V . Recent technologies enhancing the clinical utility of circulating tumor DNA. Clin Chim Acta. 2020;510:498‐506. doi:10.1016/j.cca.2020.08.010 32795543

[91] Domínguez‐Vigil IG , Moreno‐Martínez AK , Wang JY , Roehrl MHA , Barrera‐Saldaña HA . The Dawn of the liquid biopsy in the fight against cancer. Oncotarget. 2018;9(2):2912‐2922. doi:10.18632/oncotarget.23131 29416824 PMC5788692

[92] Che H , Stanley K , Jatsenko T , Thienpont B , Vermeesch JR . Expanded knowledge of cell‐free DNA biology: potential to broaden the clinical utility. Extracell Vesicles Circ Nucleic Acids. 2022;3(199–217):199‐217. doi:10.20517/evcna.2022.21 PMC11648412 39697489

[93] Huang Y‐F , Chen Y‐J , Fan T‐C , et al. Analysis of microbial sequences in plasma cell‐free DNA for early‐onset breast cancer patients and healthy females. BMC Med Genomics. 2018;11(Suppl 1):16. doi:10.1186/s12920-018-0329-y 29504912 PMC5836824

[94] Yu X , Chen H , Ji M . Epstein–Barr virus‐based nasopharyngeal carcinoma population screening. Ann Nasopharynx Cancer. 2022;6:3. doi:10.21037/anpc-21-6 PMC9392954 35996401

[95] Ji M‐F , Huang Q‐H , Yu X , et al. Evaluation of plasma Epstein–Barr virus DNA load to distinguish nasopharyngeal carcinoma patients from healthy high‐risk populations in southern China. Cancer. 2014;120(9):1353‐1360. doi:10.1002/cncr.28564 24477877

[96] Garofalo A , Alig SK , Schroers‐Martin J , et al. Viral cfDNA profiling reveals distinct EBV subtypes and stratifies risk in Hodgkin lymphomas. Blood. 2022;140(Suppl 1):1318‐1319. doi:10.1182/blood-2022-159230

[97] Bønløkke S , Stougaard M , Sorensen BS , et al. The diagnostic value of circulating cell‐free HPV DNA in plasma from cervical cancer patients. Cells. 2022;11(14):2170. doi:10.3390/cells11142170 35883612 PMC9315636

[98] Thangarajah F , Busshoff J , Salamon J , et al. Digital droplet PCR‐based quantification of ccfHPV‐DNA as liquid biopsy in HPV‐driven cervical and vulvar cancer. J Cancer Res Clin Oncol. 2023;149(14):12597‐12604. doi:10.1007/s00432-023-05077-3 37452202 PMC10587338

[99] Wang D , Hu X , Long G , Xiao L , Wang Z‐M , Zhou L‐D . The clinical value of total plasma cell‐free DNA in hepatitis B virus‐related hepatocellular carcinoma. Ann Transl Med. 2019;7(22):650. doi:10.21037/atm.2019.10.78 31930051 PMC6944585

[100] Li W , Cui X , Huo Q , et al. Profile of HBV integration in the plasma DNA of hepatocellular carcinoma patients. Curr Genomics. 2019;20(1):61‐68. doi:10.2174/1389202919666181002144336 31015792 PMC6446477

[101] Zheng B , Liu X‐L , Fan R , et al. The landscape of cell‐free HBV integrations and mutations in cirrhosis and hepatocellular carcinoma patients. Clin Cancer Res. 2021;27(13):3772‐3783. doi:10.1158/1078-0432.CCR-21-0002 33947693

[102] Polatoglou E , Mayer Z , Ungerer V , Bronkhorst AJ , Holdenrieder S . Isolation and quantification of plasma cell‐free DNA using different manual and automated methods. Diagnostics. 2022;12(10):2550. doi:10.3390/diagnostics12102550 36292239 PMC9601152

[103] Van Der Leest P , Boonstra PA , Ter Elst A , et al. Comparison of circulating cell‐free DNA extraction methods for downstream analysis in cancer patients. Cancers (Basel). 2020;12(5):1222. doi:10.3390/cancers12051222 32414097 PMC7281769

[104] Gozzetti A , Bocchia M . Liquid biopsy and blood‐based minimal residual disease evaluation in multiple myeloma. Oncol Res. 2023;31(3):271‐274. doi:10.32604/or.2023.028668 37305387 PMC10229300

[105] Devonshire AS , Whale AS , Gutteridge A , et al. Towards standardisation of cell‐free DNA measurement in plasma: controls for extraction efficiency, fragment size bias and quantification. Anal Bioanal Chem. 2014;406(26):6499‐6512. doi:10.1007/s00216-014-7835-3 24853859 PMC4182654

[106] Witt S , Neumann J , Zierdt H , Gébel G , Röscheisen C . Establishing a novel automated magnetic bead‐based method for the extraction of DNA from a variety of forensic samples. Forensic Sci Int Genet. 2012;6(5):539‐547. doi:10.1016/j.fsigen.2012.01.002 22310206

[107] Lehle S , Emons J , Hack CC , et al. Evaluation of automated techniques for extraction of circulating cell‐free DNA for implementation in standardized high‐throughput workflows. Sci Rep. 2023;13(1):373. doi:10.1038/s41598-022-27216-5 36611077 PMC9825368

[108] Jerič Kokelj B , Štalekar M , Vencken S , et al. Feasibility of droplet digital PCR analysis of plasma cell‐free DNA from kidney transplant patients. Front Med (Lausanne). 2021;8:748668. doi:10.3389/fmed.2021.748668 34692738 PMC8531215

[109] Narayan A , Carriero NJ , Gettinger SN , et al. Ultrasensitive measurement of hotspot mutations in tumor DNA in blood using error‐suppressed multiplexed deep sequencing. Cancer Res. 2012;72(14):3492‐3498. doi:10.1158/0008-5472.CAN-11-4037 22581825 PMC3426449

[110] Uchida J , Kato K , Kukita Y , et al. Diagnostic accuracy of noninvasive genotyping of EGFR in lung cancer patients by deep sequencing of plasma cell‐free DNA. Clin Chem. 2015;61(9):1191‐1196. doi:10.1373/clinchem.2015.241414 26206882

[111] Couraud S , Vaca‐Paniagua F , Villar S , et al. Noninvasive diagnosis of actionable mutations by deep sequencing of circulating free DNA in lung cancer from never‐smokers: a proof‐of‐concept study from BioCAST/IFCT‐1002. Clin Cancer Res. 2014;20(17):4613‐4624. doi:10.1158/1078-0432.CCR-13-3063 25013125

[112] Gale D , Lawson ARJ , Howarth K , et al. Development of a highly sensitive liquid biopsy platform to detect clinically‐relevant cancer mutations at Low allele fractions in cell‐free DNA. PLoS One. 2018;13(3):e0194630. doi:10.1371/journal.pone.0194630 29547634 PMC5856404

[113] Kinde I , Wu J , Papadopoulos N , Kinzler KW , Vogelstein B . Detection and quantification of rare mutations with massively parallel sequencing. Proc Natl Acad Sci. 2011;108(23):9530‐9535. doi:10.1073/pnas.1105422108 21586637 PMC3111315

[114] Tie J , Kinde I , Wang Y , et al. Circulating tumor DNA as an early marker of therapeutic response in patients with metastatic colorectal cancer. Ann Oncol. 2015;26(8):1715‐1722. doi:10.1093/annonc/mdv177 25851626 PMC4511218

[115] Belic J , Koch M , Ulz P , et al. Rapid identification of plasma DNA samples with increased ctDNA levels by a modified FAST‐SeqS approach. Clin Chem. 2015;61(6):838‐849. doi:10.1373/clinchem.2014.234286 25896989

[116] Belic J , Koch M , Ulz P , et al. mFast‐SeqS as a monitoring and pre‐screening tool for tumor‐specific aneuploidy in plasma DNA. Circulating Nucleic Acids in Serum and Plasma—CNAPS IX. In: Gahan PB , Fleischhacker M , Schmidt B , eds. Advances in Experimental Medicine and Biology. Vol 924. Springer International Publishing; 2016:147‐155. doi:10.1007/978-3-319-42044-8_28 27753036

[117] Chabon JJ , Simmons AD , Lovejoy AF , et al. Circulating tumour DNA profiling reveals heterogeneity of EGFR inhibitor resistance mechanisms in lung cancer patients. Nat Commun. 2016;7(1):11815. doi:10.1038/ncomms11815 27283993 PMC4906406

[118] Newman AM , Bratman SV , To J , et al. An ultrasensitive method for quantitating circulating tumor DNA with broad patient coverage. Nat Med. 2014;20(5):548‐554. doi:10.1038/nm.3519 24705333 PMC4016134

[119] Fredebohm J , Mehnert DH , Löber A‐K , et al. Detection and quantification of KIT mutations in ctDNA by plasma safe‐SeqS. Circulating Nucleic Acids in Serum and Plasma—CNAPS IX. In: Gahan PB , Fleischhacker M , Schmidt B , eds. Advances in Experimental Medicine and Biology. Vol 924. Springer International Publishing; 2016:187‐189. doi:10.1007/978-3-319-42044-8_34 27753042

[120] Abbosh C , Birkbak NJ , Wilson GA , et al. Phylogenetic ctDNA analysis depicts early‐stage lung cancer evolution. Nature. 2017;545(7655):446‐451. doi:10.1038/nature22364 28445469 PMC5812436

[121] Taly V , Pekin D , Benhaim L , et al. Multiplex picodroplet digital PCR to detect KRAS mutations in circulating DNA from the plasma of colorectal cancer patients. Clin Chem. 2013;59(12):1722‐1731. doi:10.1373/clinchem.2013.206359 23938455

[122] Beaver JA , Jelovac D , Balukrishna S , et al. Detection of cancer DNA in plasma of patients with early‐stage breast cancer. Clin Cancer Res. 2014;20(10):2643‐2650. doi:10.1158/1078-0432.CCR-13-2933 24504125 PMC4024333

[123] Sefrioui D , Sarafan‐Vasseur N , Beaussire L , et al. Clinical value of chip‐based digital‐PCR platform for the detection of circulating DNA in metastatic colorectal cancer. Dig Liver Dis. 2015;47(10):884‐890. doi:10.1016/j.dld.2015.05.023 26160500

[124] Taniguchi K , Uchida J , Nishino K , et al. Quantitative detection of EGFR mutations in circulating tumor DNA derived from lung adenocarcinomas. Clin Cancer Res. 2011;17(24):7808‐7815. doi:10.1158/1078-0432.CCR-11-1712 21976538

[125] Higgins MJ , Jelovac D , Barnathan E , et al. Detection of tumor PIK3CA status in metastatic breast cancer using peripheral blood. Clin Cancer Res. 2012;18(12):3462‐3469. doi:10.1158/1078-0432.CCR-11-2696 22421194 PMC3533370

[126] Li M , Diehl F , Dressman D , Vogelstein B , Kinzler KW . BEAMing up for detection and quantification of rare sequence variants. Nat Methods. 2006;3(2):95‐97. doi:10.1038/nmeth850 16432518

[127] Oxnard GR , Thress KS , Alden RS , et al. Association between plasma genotyping and outcomes of treatment with Osimertinib (AZD9291) in advanced non–small‐cell lung cancer. J Clin Oncol. 2016;34(28):3375‐3382. doi:10.1200/JCO.2016.66.7162 27354477 PMC5035123

[128] Thierry AR , Mouliere F , El Messaoudi S , et al. Clinical validation of the detection of KRAS and BRAF mutations from circulating tumor DNA. Nat Med. 2014;20(4):430‐435. doi:10.1038/nm.3511 24658074

[129] Veldore VH , Choughule A , Routhu T , et al. Validation of liquid biopsy: plasma cell‐free DNA testing in clinical management of advanced non‐small cell lung cancer. LCTT. 2018;9:1‐11. doi:10.2147/LCTT.S147841 PMC5757203 29379323

[130] Little S . Amplification‐refractory mutation system (ARMS) analysis of point mutations. Curr Protoc Hum Genet. 1995;7(1):9.8.1‐9.8.12. doi:10.1002/0471142905.hg0908s07 18428319

[131] Wang H , Jiang J , Mostert B , et al. Allele‐specific, non‐extendable primer blocker PCR (AS‐NEPB‐PCR) for DNA mutation detection in cancer. J Mol Diagn. 2013;15(1):62‐69. doi:10.1016/j.jmoldx.2012.08.007 23159590

[132] Watanabe K , Fukuhara T , Tsukita Y , et al. EGFR mutation analysis of circulating tumor DNA using an improved PNA‐LNA PCR clamp method. Can Respir J. 2016;2016:5297329. doi:10.1155/2016/5297329 27478396 PMC4961805

[133] Kim H‐R , Lee SY , Hyun D‐S , et al. Detection of EGFR mutations in circulating free DNA by PNA‐mediated PCR clamping. J Exp Clin Cancer Res. 2013;32(1):50. doi:10.1186/1756-9966-32-50 23927790 PMC3751150

[134] Miyazawa H , Tanaka T , Nagai Y , et al. Peptide nucleic acid–locked nucleic acid polymerase chain reaction clamp‐based detection test for gefitinib‐refractory T790M epidermal growth factor receptor mutation. Cancer Sci. 2008;99(3):595‐600. doi:10.1111/j.1349-7006.2007.00706.x 18271876 PMC11158388

[135] Freidin MB , Freydina DV , Leung M , Montero Fernandez A , Nicholson AG , Lim E . Circulating tumor DNA outperforms circulating tumor cells for KRAS mutation detection in thoracic malignancies. Clin Chem. 2015;61(10):1299‐1304. doi:10.1373/clinchem.2015.242453 26272233

[136] Mastoraki S , Strati A , Tzanikou E , et al. ESR1 methylation: a liquid biopsy–based epigenetic assay for the follow‐up of patients with metastatic breast cancer receiving endocrine treatment. Clin Cancer Res. 2018;24(6):1500‐1510. doi:10.1158/1078-0432.CCR-17-1181 29284708

[137] Elazezy M , Joosse SA . Techniques of using circulating tumor DNA as a liquid biopsy component in cancer management. Comput Struct Biotechnol J. 2018;16:370‐378. doi:10.1016/j.csbj.2018.10.002 30364656 PMC6197739

[138] Arasawa S , Takeda H , Takai A , et al. Evolutional transition of HBV genome during the persistent infection determined by single‐molecule real‐time sequencing. Hepatol Commun. 2023;7(3):e0047. doi:10.1097/HC9.0000000000000047 36848123 PMC9974078

[139] Yu SCY , Deng J , Qiao R , et al. Comparison of single molecule, real‐time sequencing and nanopore sequencing for analysis of the size, end‐motif, and tissue‐of‐origin of long cell‐free DNA in plasma. Clin Chem. 2023;69(2):168‐179. doi:10.1093/clinchem/hvac180 36322427

[140] Xia Y , Rao R , Xiong M , et al. CRISPR‐powered strategies for amplification‐free diagnostics of infectious diseases. Anal Chem. 2024;96(20):8091‐8108. doi:10.1021/acs.analchem.3c04363 38451204

[141] Cisneros‐Villanueva M , Hidalgo‐Pérez L , Rios‐Romero M , et al. Cell‐free DNA analysis in current cancer clinical trials: a review. Br J Cancer. 2022;126(3):391‐400. doi:10.1038/s41416-021-01696-0 35027672 PMC8810765

[142] Sultana GNN , Akter F , Israfil SMH , et al. Quantitative analysis of serum cell‐free DNA as a predictive and prognostic marker in breast cancer patients. Front Oncol. 2023;13:1171412. doi:10.3389/fonc.2023.1171412 37427131 PMC10324030

[143] Crucitta S , Ruglioni M , Novi C , et al. Comparison of digital PCR systems for the analysis of liquid biopsy samples of patients affected by lung and colorectal cancer. Clin Chim Acta. 2023;541:117239. doi:10.1016/j.cca.2023.117239 36736684

[144] Sorbini M , Carradori T , Togliatto GM , Vaisitti T , Deaglio S . Technical advances in circulating cell‐free DNA detection and analysis for personalized medicine in patients' Care. Biomolecules. 2024;14(4):498. doi:10.3390/biom14040498 38672514 PMC11048502

[145] Schwarz UI , Gulilat M , Kim RB . The role of next‐generation sequencing in pharmacogenetics and pharmacogenomics. Cold Spring Harb Perspect Med. 2019;9(2):a033027. doi:10.1101/cshperspect.a033027 29844222 PMC6360866

[146] Branton D , Deamer DW , Marziali A , et al. The potential and challenges of nanopore sequencing. Nat Biotechnol. 2008;26(10):1146‐1153. doi:10.1038/nbt.1495 18846088 PMC2683588

[147] Kaminski MM , Abudayyeh OO , Gootenberg JS , Zhang F , Collins JJ . CRISPR‐based diagnostics. Nat Biomed Eng. 2021;5(7):643‐656. doi:10.1038/s41551-021-00760-7 34272525

[148] Freije CA , Myhrvold C , Boehm CK , et al. Programmable inhibition and detection of RNA viruses using Cas13. Mol Cell. 2019;76(5):826‐837.e11. doi:10.1016/j.molcel.2019.09.013 31607545 PMC7422627

[149] Zhang X , Li J , Zhuang Z , Wang J , Bu Z , Lan X . Challenges and prospects of cell‐free DNA in precision oncology. Medicine Plus. 2024;1(4):100059. doi:10.1016/j.medp.2024.100059

[150] Li S , Zeng W , Ni X , et al. Comprehensive tissue deconvolution of cell‐free DNA by deep learning for disease diagnosis and monitoring. Proc Natl Acad Sci U S A. 2023;120(28):e2305236120. doi:10.1073/pnas.2305236120 37399400 PMC10334733

[151] Li Y , Xu J , Chen C , et al. Multimodal epigenetic sequencing analysis (MESA) of cell‐free DNA for non‐invasive colorectal cancer detection. Genome Med. 2024;16(1):9. doi:10.1186/s13073-023-01280-6 38225592 PMC10790422

[152] Martin‐Alonso C , Tabrizi S , Xiong K , et al. Priming agents transiently reduce the clearance of cell‐free DNA to improve liquid biopsies. Science. 2024;383(6680):eadf2341. doi:10.1126/science.adf2341 38236959 PMC11529396

[153] Esfahani MS , Hamilton EG , Mehrmohamadi M , et al. Inferring gene expression from cell‐free DNA fragmentation profiles. Nat Biotechnol. 2022;40(4):585‐597. doi:10.1038/s41587-022-01222-4 35361996 PMC9337986

[154] Song P , Wu LR , Yan YH , et al. Limitations and opportunities of technologies for the analysis of cell‐free DNA in cancer diagnostics. Nat Biomed Eng. 2022;6(3):232‐245. doi:10.1038/s41551-021-00837-3 35102279 PMC9336539

[155] Agashe R , Kurzrock R . Circulating tumor cells: from the laboratory to the cancer clinic. Cancers (Basel). 2020;12(9):2361. doi:10.3390/cancers12092361 32825548 PMC7564158

[156] Tewari KS , Sill MW , Monk BJ , et al. Circulating tumor cells in advanced cervical cancer: NRG oncology—gynecologic oncology group study 240 (NCT 00803062). Mol Cancer Ther. 2020;19(11):2363‐2370. doi:10.1158/1535-7163.MCT-20-0276 32847980 PMC7907274

[157] He C , Huang X , Su X , et al. The association between circulating tumor cells and Epstein–Barr virus activation in patients with nasopharyngeal carcinoma. Cancer Biol Ther. 2017;18(11):888‐894. doi:10.1080/15384047.2017.1281493 28121221 PMC5710676

[158] Chang Y , Jeong SW , Jang JY , et al. The diagnostic value of circulating tumor DNA in hepatitis B virus induced hepatocellular carcinoma: a systematic review and meta‐analysis. J Liver Cancer. 2022;22(2):167‐177. doi:10.17998/jlc.2022.09.19 37383408 PMC10035733

[159] Janjua D , Chaudhary A , Joshi U , Tripathi T , Bharti AC . Circulating tumor cells in solid malignancies: from advanced isolation technologies to biological understanding and clinical relevance in early diagnosis and prognosis. Biochim Biophys Acta Rev Cancer. 2025;1880(1):189236. doi:10.1016/j.bbcan.2024.189236 39662757

[160] Phung Q , Lin MJ , Xie H , Greninger AL . Fragment size‐based enrichment of viral sequences in plasma cell‐free DNA. J Mol Diagn. 2022;24(5):476‐484. doi:10.1016/j.jmoldx.2022.01.007 35569878 PMC9127460

[161] Kanakry JA , Hegde AM , Durand CM , et al. The clinical significance of EBV DNA in the plasma and peripheral blood mononuclear cells of patients with or without EBV diseases. Blood. 2016;127(16):2007‐2017. doi:10.1182/blood-2015-09-672030 26744460 PMC4841041

[162] Shamay M , Kanakry JA , Low JSW , et al. CpG methylation in cell‐free Epstein–Barr virus DNA in patients with EBV‐Hodgkin lymphoma. Blood Adv. 2020;4(8):1624‐1627. doi:10.1182/bloodadvances.2020001511 32311011 PMC7189298

[163] Krasniqi E , Barba M , Venuti A , et al. Circulating HPV DNA in the management of oropharyngeal and cervical cancers: current knowledge and future perspectives. J Clin Med. 2021;10(7):1525. doi:10.3390/jcm10071525 33917435 PMC8038737

[164] Kataria R , Shoaie S , Grigoriadis A , Wan JCM . Leveraging circulating microbial DNA for early cancer detection. Trends Cancer. 2023;9(11):879‐882. doi:10.1016/j.trecan.2023.08.001 37659908 PMC10873208

[165] Pascual J , Attard G , Bidard F‐C , et al. ESMO recommendations on the use of circulating tumour DNA assays for patients with cancer: a report from the ESMO precision medicine working group. Ann Oncol. 2022;33(8):750‐768. doi:10.1016/j.annonc.2022.05.520 35809752

[166] Fiala C , Diamandis EP . New approaches for detecting cancer with circulating cell‐free DNA. BMC Med. 2019;17(1):159. doi:10.1186/s12916-019-1400-z 31416458 PMC6696683

[167] Bettegowda C , Sausen M , Leary RJ , et al. Detection of circulating tumor DNA in early‐ and late‐stage human malignancies. Sci Transl Med. 2014:224ra24. doi:10.1126/scitranslmed.3007094 PMC4017867 24553385

[168] Reed SC , Croessmann S , Park BH . CHIP happens: clonal hematopoiesis of indeterminate potential and its relationship to solid tumors. Clin Cancer Res. 2023;29(8):1403‐1411. doi:10.1158/1078-0432.CCR-22-2598 36454121 PMC10106364

[169] Dondorp W . Towards responsible ctDNA‐based multi‐cancer screening: a preliminary exploration and discussion of ethically relevant aspects. Extracell Vesicles Circ Nucleic Acids. 2022;2(3):218‐226. doi:10.20517/evcna.2022.23 PMC11648474 39697487

[170] Hall A , Bostanci A , Wright CF . Non‐invasive prenatal diagnosis using cell‐free fetal DNA technology: applications and implications. Public Health Genomics. 2010;13(4):246‐255. doi:10.1159/000279626 20395693

[171] Nahvijou A , Hadji M , Marnani AB , et al. A systematic review of economic aspects of cervical cancer screening strategies worldwide: discrepancy between economic analysis and policymaking. Asian Pac J Cancer Prev. 2014;15(19):8229‐8237. 25339011

[172] Kuhlmann A , Weiss GJ , Beck J , et al. Cost‐minimization analysis of using tumor cell‐free DNA as monitoring tool in cancer immunotherapy. JCO. 2019;37(15_suppl):6642. doi:10.1200/JCO.2019.37.15_suppl.6642

[173] Liu L , Ying C , Zhao Z , et al. Identification of reliable biomarkers of human papillomavirus 16 methylation in cervical lesions based on integration status using high‐resolution melting analysis. Clin Epigenetics. 2018;10(1):10. doi:10.1186/s13148-018-0445-8 29410710 PMC5781301

[174] Moser T , Kühberger S , Lazzeri I , Vlachos G , Heitzer E . Bridging biological cfDNA features and machine learning approaches. Trends Genet. 2023;39(4):285‐307. doi:10.1016/j.tig.2023.01.004 36792446

[175] Yin Z , Feng T , Xu Q , et al. Monitoring of cell‐free human papillomavirus DNA in metastatic or recurrent cervical cancer: clinical significance and treatment implications. Elife. 2024;13:RP101887. doi:10.7554/eLife.101887.1 PMC12443474 40960177

[176] Turning the tide of early cancer detection. Nat Med. 2024;30(5):1217. doi:10.1038/s41591-024-03046-y 38773344

[177] Nguyen TH , Doan NNT , Tran TH , et al. Tissue of origin detection for cancer tumor using low‐depth cfDNA samples through combination of tumor‐specific methylation atlas and genome‐wide methylation density in graph convolutional neural networks. J Transl Med. 2024;22(1):618. doi:10.1186/s12967-024-05416-z 38961476 PMC11223394

